# Selecting women for breast cancer chemoprevention and what agents should be used

**DOI:** 10.1186/1897-4287-10-S2-A10

**Published:** 2012-04-12

**Authors:** J Cuzick

**Affiliations:** 1 Centre for Cancer Prevention, Wolfson Institute of Preventive Medicine, Queen Mary University of London, Charterhouse Square, London EC1M 6BQ, UK

## 

Recent evidence on the chemoprevention of breast cancer has come from a number of sources:

1) Recent data on two new SERMs – lasofoxifene and arzoxifene– suggests they might be an attractive option, but further details are needed to fully evaluate their role.

2) Update on the tamoxifen and raloxifene preventive trials have shown a continued benefit after treatment completion, and an overview is ongoing to combine all the post-treatment data on risks and benefits. Latest results from the STAR trial indicate that raloxifene is less efficacious than tamoxifen but has fewer side effects. A full new overview is in progress.

3) A report from the ATAC trial indicating that the occurrence of endocrine symptoms predicts response to both tamoxifen and anastrozole may also have relevance in the preventive setting.

4) Long term follow-up of the adjuvant trials using aromatase trials has provided new evidence on the duration of a potential preventive effect by looking at isolated new contralateral tumours. This has been an excellent model for tamoxifen and may well also be so for the AIs.

5) A very recent report from the MAP3 (Goss, NEJM,2011) indicates that after 35 months median follow up , a 60% reduction in new tumours was seen , confirming predictions made from contralateral tumours in adjuvant trials.

6) New data confirm the long term protective effect of aspirin on breast cancer, but suggests it needs to be taken for at least 5 years to get a benefit. Mixed data have appeared for the statins.
